# Supplementary material for: Hot spot prediction in protein-protein interactions by an ensemble system
Source: BMC Syst Biol. 2018 Dec 31;12(Suppl 9):132. doi: 10.1186/s12918-018-0665-8 (PMC6311905; doi:10.1186/s12918-018-0665-8)
Supplement: Supplementary file 2 — All relevant MATLAB code. A simple Matlab implement of our predictor. (ZIP 19,742 kb) [file 12918_2018_665_MOESM2_ESM.zip › Additional file 2/Read ASA/ASA website/dbMPIKT/2NYYD.html]

 NetSurfP - Protein Surface Accessibility and Secondary Structure Predictions

  

|  |  |  |
| --- | --- | --- |
|  |  | NetSurfP - Protein Surface Accessibility and Secondary Structure Predictions  Technical University of Denmark |

  


---

```
# For publication of results, please cite:
# A generic method for assignment of reliability scores applied to solvent accessibility predictions.
# Bent Petersen, Thomas Nordahl Petersen, Pernille Andersen, Morten Nielsen and Claus Lundegaard
# BMC Structural Biology 2009, 9:51 doi:10.1186/1472-6807-9-51
#
# Column 1: Class assignment - B for buried or E for Exposed - Threshold: 25% exposure, but not based on RSA
# Column 2: Amino acid
# Column 3: Sequence name
# Column 4: Amino acid number
# Column 5: Relative Surface Accessibility - RSA
# Column 6: Absolute Surface Accessibility
# Column 7: Z-fit score for RSA prediction
# Column 8: Probability for Alpha-Helix
# Column 9: Probability for Beta-strand
# Column 10: Probability for Coil
E Q  2NYYD                  1    0.754 134.629   1.159   0.005   0.015   0.979
B V  2NYYD                  2    0.213  32.800  -0.235   0.003   0.718   0.279
E Q  2NYYD                  3    0.432  77.119   1.294   0.001   0.900   0.099
B L  2NYYD                  4    0.154  28.252   0.680   0.002   0.816   0.182
E Q  2NYYD                  5    0.379  67.689   0.494   0.003   0.718   0.279
B E  2NYYD                  6    0.240  41.911   0.577   0.004   0.514   0.481
E S  2NYYD                  7    0.423  49.576  -0.508   0.004   0.197   0.799
E G  2NYYD                  8    0.496  39.051  -1.862   0.018   0.088   0.893
E G  2NYYD                  9    0.525  41.357  -2.331   0.018   0.047   0.935
E G  2NYYD                 10    0.514  40.436  -1.768   0.020   0.205   0.775
E L  2NYYD                 11    0.402  73.661  -0.813   0.022   0.359   0.619
E V  2NYYD                 12    0.287  44.189   0.636   0.021   0.451   0.528
E Q  2NYYD                 13    0.425  75.941   1.144   0.020   0.205   0.775
E P  2NYYD                 14    0.459  65.090  -0.558   0.018   0.047   0.935
E G  2NYYD                 15    0.599  47.157  -2.089   0.005   0.045   0.951
E G  2NYYD                 16    0.416  32.708  -0.687   0.004   0.138   0.858
E S  2NYYD                 17    0.427  50.056  -0.574   0.004   0.420   0.576
B L  2NYYD                 18    0.159  29.058   0.749   0.002   0.816   0.182
E R  2NYYD                 19    0.425  97.302   0.858   0.001   0.900   0.099
B L  2NYYD                 20    0.077  14.025   0.585   0.001   0.900   0.099
B S  2NYYD                 21    0.174  20.416   0.726   0.001   0.900   0.099
B C  2NYYD                 22    0.047   6.599   0.123   0.001   0.900   0.099
B A  2NYYD                 23    0.293  32.245   0.508   0.001   0.900   0.099
B A  2NYYD                 24    0.086   9.532  -0.432   0.002   0.816   0.182
B S  2NYYD                 25    0.177  20.768  -0.619   0.004   0.514   0.481
B G  2NYYD                 26    0.250  19.644  -1.648   0.004   0.197   0.799
B F  2NYYD                 27    0.243  48.770  -0.832   0.005   0.336   0.660
B T  2NYYD                 28    0.200  27.685  -0.751   0.004   0.514   0.481
B F  2NYYD                 29    0.113  22.759  -0.708   0.021   0.451   0.528
B K  2NYYD                 30    0.233  47.908  -1.119   0.021   0.279   0.699
B Y  2NYYD                 31    0.287  61.396  -1.123   0.018   0.088   0.893
B D  2NYYD                 32    0.112  16.139  -0.395   0.019   0.141   0.840
B Y  2NYYD                 33    0.091  19.383  -0.027   0.022   0.359   0.619
B M  2NYYD                 34    0.029   5.763   0.161   0.023   0.655   0.322
B Y  2NYYD                 35    0.048  10.215  -0.102   0.021   0.756   0.223
B W  2NYYD                 36    0.035   8.466   0.430   0.021   0.756   0.223
B V  2NYYD                 37    0.036   5.502   0.446   0.018   0.846   0.136
B R  2NYYD                 38    0.109  24.984   0.484   0.023   0.655   0.322
B Q  2NYYD                 39    0.088  15.735   0.330   0.022   0.359   0.619
B A  2NYYD                 40    0.198  21.875   0.728   0.005   0.045   0.951
E P  2NYYD                 41    0.355  50.318   0.723   0.005   0.015   0.979
E G  2NYYD                 42    0.574  45.213  -1.683   0.018   0.019   0.964
E K  2NYYD                 43    0.383  78.845   0.573   0.018   0.019   0.964
E G  2NYYD                 44    0.425  33.424  -0.428   0.018   0.088   0.893
B L  2NYYD                 45    0.162  29.754   0.925   0.022   0.359   0.619
B E  2NYYD                 46    0.096  16.736  -0.156   0.004   0.616   0.381
B W  2NYYD                 47    0.044  10.630   0.070   0.001   0.900   0.099
B V  2NYYD                 48    0.024   3.673   0.892   0.001   0.959   0.040
B A  2NYYD                 49    0.018   1.928  -0.046   0.001   0.959   0.040
B T  2NYYD                 50    0.075  10.347  -0.386   0.001   0.959   0.040
B I  2NYYD                 51    0.023   4.163   0.975   0.001   0.959   0.040
B S  2NYYD                 52    0.144  16.889   0.664   0.002   0.816   0.182
B D  2NYYD                 53    0.266  38.316  -0.945   0.005   0.336   0.660
E G  2NYYD                 54    0.540  42.537  -1.809   0.005   0.015   0.979
E G  2NYYD                 55    0.540  42.459  -2.563   0.003   0.003   0.994
E S  2NYYD                 56    0.541  63.370  -2.288   0.018   0.019   0.964
E Y  2NYYD                 57    0.477 101.978  -0.483   0.018   0.047   0.935
E T  2NYYD                 58    0.308  42.733  -0.026   0.019   0.141   0.840
E Y  2NYYD                 59    0.427  91.271   0.031   0.020   0.205   0.775
B Y  2NYYD                 60    0.198  42.377   0.394   0.020   0.205   0.775
B S  2NYYD                 61    0.203  23.792  -0.503   0.052   0.084   0.864
E D  2NYYD                 62    0.632  91.028  -0.690   0.053   0.043   0.903
E S  2NYYD                 63    0.410  48.075  -0.023   0.018   0.047   0.935
B V  2NYYD                 64    0.081  12.496  -0.081   0.019   0.141   0.840
E E  2NYYD                 65    0.393  68.675   0.784   0.019   0.141   0.840
B G  2NYYD                 66    0.217  17.102  -1.511   0.005   0.262   0.733
B R  2NYYD                 67    0.204  46.693   0.776   0.003   0.718   0.279
B F  2NYYD                 68    0.051  10.135  -0.163   0.001   0.900   0.099
B T  2NYYD                 69    0.101  13.981   0.531   0.001   0.959   0.040
B T  2NYYD                 70    0.041   5.645   0.564   0.001   0.959   0.040
B S  2NYYD                 71    0.081   9.435   0.187   0.002   0.816   0.182
B R  2NYYD                 72    0.247  56.517   0.586   0.004   0.616   0.381
B D  2NYYD                 73    0.214  30.794  -0.460   0.004   0.420   0.576
E N  2NYYD                 74    0.425  62.235  -0.252   0.018   0.088   0.893
B S  2NYYD                 75    0.237  27.835  -1.884   0.018   0.047   0.935
E K  2NYYD                 76    0.508 104.434   0.640   0.018   0.088   0.893
B N  2NYYD                 77    0.244  35.648  -0.632   0.022   0.359   0.619
B T  2NYYD                 78    0.188  26.020   0.774   0.018   0.846   0.136
B L  2NYYD                 79    0.069  12.542  -0.535   0.011   0.918   0.071
B Y  2NYYD                 80    0.160  34.107   0.586   0.006   0.962   0.032
B L  2NYYD                 81    0.042   7.690   0.916   0.011   0.918   0.071
B Q  2NYYD                 82    0.152  27.111  -0.134   0.011   0.918   0.071
B M  2NYYD                 83    0.060  11.966  -0.554   0.023   0.655   0.322
E N  2NYYD                 84    0.359  52.602   0.025   0.021   0.451   0.528
E S  2NYYD                 85    0.355  41.653  -0.930   0.020   0.205   0.775
B L  2NYYD                 86    0.141  25.872   0.077   0.019   0.141   0.840
E R  2NYYD                 87    0.478 109.439   0.847   0.018   0.088   0.893
E A  2NYYD                 88    0.390  42.967  -0.563   0.018   0.088   0.893
E E  2NYYD                 89    0.435  76.064  -0.786   0.053   0.043   0.903
B D  2NYYD                 90    0.216  31.082  -0.825   0.018   0.088   0.893
B T  2NYYD                 91    0.099  13.787   0.209   0.022   0.359   0.619
B A  2NYYD                 92    0.033   3.604  -0.359   0.018   0.846   0.136
B I  2NYYD                 93    0.057  10.453   0.599   0.011   0.918   0.071
B Y  2NYYD                 94    0.030   6.411   0.890   0.011   0.918   0.071
B Y  2NYYD                 95    0.047  10.065   0.782   0.018   0.846   0.136
B C  2NYYD                 96    0.016   2.246   1.428   0.021   0.756   0.223
B S  2NYYD                 97    0.028   3.317  -0.096   0.023   0.655   0.322
B R  2NYYD                 98    0.132  30.251  -0.253   0.022   0.359   0.619
E Y  2NYYD                 99    0.309  66.055  -0.716   0.020   0.205   0.775
E R  2NYYD                100    0.303  69.387  -0.488   0.018   0.088   0.893
E Y  2NYYD                101    0.361  77.060  -1.688   0.018   0.088   0.893
E D  2NYYD                102    0.420  60.450  -1.860   0.018   0.088   0.893
B D  2NYYD                103    0.283  40.824  -1.172   0.018   0.088   0.893
B A  2NYYD                104    0.175  19.230  -1.265   0.018   0.088   0.893
B M  2NYYD                105    0.078  15.528  -0.099   0.019   0.141   0.840
B D  2NYYD                106    0.106  15.217  -0.689   0.019   0.141   0.840
B Y  2NYYD                107    0.086  18.421  -0.370   0.019   0.141   0.840
B W  2NYYD                108    0.050  12.121  -0.852   0.019   0.141   0.840
B G  2NYYD                109    0.046   3.589  -1.940   0.019   0.141   0.840
B Q  2NYYD                110    0.169  30.255  -0.407   0.004   0.085   0.910
B G  2NYYD                111    0.157  12.356  -1.712   0.004   0.138   0.858
B T  2NYYD                112    0.080  11.054  -0.334   0.005   0.336   0.660
E L  2NYYD                113    0.263  48.155  -0.187   0.003   0.718   0.279
B V  2NYYD                114    0.045   6.932   0.262   0.001   0.900   0.099
B T  2NYYD                115    0.151  20.902   0.194   0.001   0.900   0.099
B V  2NYYD                116    0.061   9.437   0.228   0.002   0.816   0.182
B S  2NYYD                117    0.168  19.725  -0.511   0.004   0.514   0.481
B S  2NYYD                118    0.184  21.541  -1.554   0.004   0.197   0.799
E A  2NYYD                119    0.460  50.659  -1.661   0.018   0.088   0.893
E S  2NYYD                120    0.509  59.655  -0.590   0.018   0.047   0.935
E T  2NYYD                121    0.449  62.276  -1.612   0.018   0.047   0.935
E K  2NYYD                122    0.607 124.942  -1.737   0.018   0.019   0.964
E G  2NYYD                123    0.488  38.406  -1.844   0.018   0.019   0.964
E P  2NYYD                124    0.404  57.285  -1.958   0.018   0.047   0.935
E S  2NYYD                125    0.486  56.924  -1.251   0.020   0.205   0.775
E V  2NYYD                126    0.317  48.738  -1.591   0.022   0.359   0.619
B F  2NYYD                127    0.151  30.326   0.560   0.021   0.279   0.699
E P  2NYYD                128    0.343  48.686  -0.568   0.020   0.205   0.775
B L  2NYYD                129    0.151  27.703  -0.793   0.020   0.205   0.775
E A  2NYYD                130    0.331  36.432  -0.916   0.004   0.138   0.858
E P  2NYYD                131    0.317  45.011  -1.878   0.004   0.085   0.910
B S  2NYYD                132    0.203  23.803  -1.967   0.005   0.045   0.951
E S  2NYYD                133    0.422  49.505  -2.640   0.005   0.045   0.951
E K  2NYYD                134    0.616 126.691  -2.381   0.005   0.015   0.979
E S  2NYYD                135    0.377  44.149  -2.497   0.005   0.015   0.979
E T  2NYYD                136    0.460  63.760  -1.403   0.005   0.015   0.979
E S  2NYYD                137    0.498  58.412  -1.356   0.005   0.015   0.979
E G  2NYYD                138    0.637  50.116  -2.569   0.005   0.015   0.979
E G  2NYYD                139    0.452  35.596  -1.730   0.005   0.045   0.951
E T  2NYYD                140    0.381  52.775   0.221   0.004   0.420   0.576
B A  2NYYD                141    0.066   7.317   0.227   0.002   0.816   0.182
B A  2NYYD                142    0.200  22.040   0.469   0.001   0.959   0.040
B L  2NYYD                143    0.047   8.661  -0.223   0.001   0.959   0.040
B G  2NYYD                144    0.044   3.463  -0.371   0.001   0.959   0.040
B C  2NYYD                145    0.030   4.156  -0.039   0.001   0.900   0.099
B L  2NYYD                146    0.161  29.479   0.504   0.001   0.900   0.099
B V  2NYYD                147    0.063   9.745  -0.574   0.003   0.718   0.279
B K  2NYYD                148    0.285  58.707  -0.130   0.004   0.420   0.576
E D  2NYYD                149    0.437  62.928  -1.483   0.005   0.262   0.733
B Y  2NYYD                150    0.220  47.014  -0.794   0.005   0.336   0.660
E F  2NYYD                151    0.313  62.739  -0.238   0.005   0.262   0.733
B P  2NYYD                152    0.229  32.509  -1.062   0.004   0.197   0.799
E E  2NYYD                153    0.537  93.831  -0.649   0.004   0.138   0.858
E P  2NYYD                154    0.370  52.475  -1.622   0.004   0.138   0.858
B V  2NYYD                155    0.167  25.729  -0.019   0.003   0.718   0.279
B T  2NYYD                156    0.262  36.409  -0.306   0.001   0.900   0.099
B V  2NYYD                157    0.052   8.023   0.241   0.001   0.959   0.040
B S  2NYYD                158    0.211  24.682   0.234   0.011   0.918   0.071
B W  2NYYD                159    0.067  16.065   0.028   0.011   0.918   0.071
E N  2NYYD                160    0.336  49.234   0.163   0.002   0.816   0.182
E S  2NYYD                161    0.463  54.287  -0.723   0.005   0.336   0.660
E G  2NYYD                162    0.488  38.429  -0.994   0.018   0.047   0.935
E A  2NYYD                163    0.597  65.789  -0.519   0.005   0.015   0.979
E L  2NYYD                164    0.475  86.881  -0.451   0.005   0.015   0.979
E T  2NYYD                165    0.565  78.310  -2.306   0.005   0.015   0.979
E S  2NYYD                166    0.610  71.515  -0.667   0.005   0.015   0.979
E G  2NYYD                167    0.440  34.659  -0.657   0.005   0.045   0.951
B V  2NYYD                168    0.111  16.984  -1.165   0.005   0.262   0.733
E H  2NYYD                169    0.388  70.559   0.247   0.003   0.718   0.279
E T  2NYYD                170    0.334  46.312  -0.654   0.002   0.816   0.182
B F  2NYYD                171    0.190  38.053   1.165   0.004   0.616   0.381
B P  2NYYD                172    0.142  20.192  -0.102   0.022   0.359   0.619
B A  2NYYD                173    0.216  23.836  -1.180   0.023   0.655   0.322
B V  2NYYD                174    0.231  35.520  -0.620   0.002   0.816   0.182
B L  2NYYD                175    0.236  43.230  -0.491   0.003   0.718   0.279
E Q  2NYYD                176    0.465  83.031  -0.789   0.004   0.420   0.576
E S  2NYYD                177    0.668  78.278  -2.521   0.004   0.085   0.910
E S  2NYYD                178    0.631  73.965  -2.061   0.005   0.045   0.951
E G  2NYYD                179    0.509  40.058  -1.960   0.004   0.085   0.910
E L  2NYYD                180    0.410  75.034  -0.380   0.004   0.420   0.576
B Y  2NYYD                181    0.269  57.485  -1.184   0.004   0.616   0.381
B S  2NYYD                182    0.161  18.869  -1.719   0.003   0.718   0.279
B L  2NYYD                183    0.190  34.734  -1.269   0.002   0.816   0.182
B S  2NYYD                184    0.150  17.615  -0.515   0.001   0.900   0.099
B S  2NYYD                185    0.067   7.794  -1.278   0.001   0.900   0.099
B V  2NYYD                186    0.142  21.749   0.343   0.001   0.900   0.099
B V  2NYYD                187    0.114  17.491  -0.613   0.001   0.900   0.099
B T  2NYYD                188    0.200  27.740   0.619   0.002   0.816   0.182
B V  2NYYD                189    0.118  18.075   0.775   0.003   0.718   0.279
E P  2NYYD                190    0.348  49.452  -0.038   0.021   0.451   0.528
E S  2NYYD                191    0.352  41.254  -0.603   0.020   0.205   0.775
E S  2NYYD                192    0.543  63.581  -0.254   0.021   0.279   0.699
E S  2NYYD                193    0.671  78.629   0.463   0.020   0.205   0.775
B L  2NYYD                194    0.252  46.123  -0.833   0.018   0.088   0.893
E G  2NYYD                195    0.738  58.049   0.444   0.005   0.015   0.979
E T  2NYYD                196    0.699  96.951   0.676   0.005   0.015   0.979
E Q  2NYYD                197    0.495  88.407   0.782   0.005   0.262   0.733
E T  2NYYD                198    0.491  68.157   1.358   0.002   0.816   0.182
B Y  2NYYD                199    0.070  14.874   0.847   0.001   0.959   0.040
E I  2NYYD                200    0.314  58.182   1.949   0.001   0.959   0.040
B C  2NYYD                201    0.023   3.271   0.580   0.001   0.959   0.040
E N  2NYYD                202    0.328  48.063   0.941   0.001   0.900   0.099
B V  2NYYD                203    0.079  12.081  -0.658   0.001   0.900   0.099
E N  2NYYD                204    0.523  76.582   0.478   0.002   0.816   0.182
B H  2NYYD                205    0.179  32.578  -0.048   0.004   0.514   0.481
E K  2NYYD                206    0.578 118.977  -0.157   0.005   0.045   0.951
E P  2NYYD                207    0.741 105.134  -1.903   0.005   0.015   0.979
E S  2NYYD                208    0.563  65.937  -0.779   0.005   0.045   0.951
E N  2NYYD                209    0.560  81.999  -0.504   0.004   0.085   0.910
E T  2NYYD                210    0.476  66.077   0.369   0.005   0.336   0.660
E K  2NYYD                211    0.550 113.073   1.179   0.003   0.718   0.279
B V  2NYYD                212    0.104  15.939   0.074   0.002   0.816   0.182
E D  2NYYD                213    0.624  89.962  -0.227   0.001   0.900   0.099
E K  2NYYD                214    0.252  51.754   0.708   0.002   0.816   0.182
E K  2NYYD                215    0.570 117.270   0.459   0.003   0.718   0.279
B V  2NYYD                216    0.174  26.682   0.350   0.021   0.451   0.528
E E  2NYYD                217    0.523  91.386  -0.025   0.004   0.197   0.799
E P  2NYYD                218    0.583  82.714  -1.625   0.004   0.138   0.858
E K  2NYYD                219    0.556 114.369  -0.090   0.004   0.085   0.910
E S  2NYYD                220    0.442  51.861  -1.246   0.005   0.045   0.951
E C  2NYYD                221    0.393  55.219  -1.675   0.018   0.019   0.964
E D  2NYYD                222    0.682  98.319  -2.603   0.018   0.019   0.964
E K  2NYYD                223    0.768 158.019   0.223   0.003   0.003   0.994

Explain the output.  Go back.
```

---
